# Ovarian cancer targeted therapy: current landscape and future challenges

**DOI:** 10.3389/fonc.2025.1535235

**Published:** 2025-05-06

**Authors:** Guangli Wang, Hui Yang, Yuling Wang, Juan Qin

**Affiliations:** ^1^ Yanbian University Affiliated Hospital Obstetrics and Gynecology Department, Yanbian, China; ^2^ Clinical Medicine School of Guizhou Medical University, Guiyang, China; ^3^ The Maternal and Child Health Care Hospital of Guizhou Medical University, Guiyang, China

**Keywords:** ovarian cancer, targeted therapy, combination therapy, personalized medicine, adverse reactions

## Abstract

Ovarian cancer is one of the deadliest malignancies in women and remains the leading cause of death from gynecological cancers worldwide. The standard treatment typically involves tumor-reducing surgery and cytotoxic chemotherapy; however, many patients are unable to tolerate the side effects of these treatments or experience recurrence due to significant drug resistance, which limits the overall clinical benefits. Consequently, there is a pressing need for novel therapeutic strategies. In recent years, Targeted therapies, including anti-angiogenic drugs, PARP inhibitors, and immune checkpoint inhibitors, have revolutionized ovarian cancer treatment. Additionally, drug targeting and therapeutic efficacy have been substantially enhanced through carrier technologies and conjugation strategies, such as antibody-drug conjugates, polymer-drug conjugates, and dual-targeted nanomedicines. These innovative strategies aim to selectively target ovarian cancer cells, overcome drug resistance, and reduce systemic toxicity, thus achieving optimal therapeutic outcomes. This review aims to critically evaluate the progress and challenges in ovarian cancer targeted therapy and propose future research directions to improve clinical outcomes efforts toward providing more effective and personalized treatment options for ovarian cancer patients.

## 1 Introduction

Ovarian cancer is one of the leading causes of cancer-related deaths in women. Due to the lack of prominent early symptoms, more than 70% of patients are diagnosed at advanced stages (III or IV) (1). Epithelial ovarian cancer (EOC) is the most common type, accounting for 90% of all ovarian cancer cases, with high-grade serous ovarian cancer (HGSOC) being the most aggressive and recurrent subtype (2). The standard treatments for ovarian cancer include cytoreductive surgery and platinum-based chemotherapy. Over 80% of newly diagnosed patients achieve partial or complete remission after initial treatment; however, most experience relapse within three years, and treatment outcomes after relapse are poor due to significant drug resistance (3). Managing platinum-resistant ovarian cancer is challenging due to limited treatment options and poor survival outcomes for recurrent patients (4–6). Consequently, identifying novel strategies to overcome resistance and extend patient survival has become a critical research focus (7). In recent years, molecular studies have provided greater insight into the mechanisms driving ovarian cancer, Targeted therapies have emerged as a promising approach, aiming to improve treatment efficacy while minimizing toxicity to healthy tissues by targeting key molecules involved in tumor growth and progression. Anti-angiogenesis inhibitors (8), PARP inhibitors (PARPis) (9), and immune checkpoint inhibitors (ICIs) (10) have shown promising results in clinical trials, with some drugs already approved for treating specific ovarian cancer subtypes. Furthermore, small-molecule tyrosine kinase inhibitors, lipid metabolism-targeting agents, and gene therapy are also being actively investigated (11).This review focuses on the latest advances in targeted therapies approved for ovarian cancer, particularly innovations in combination treatment strategies integrating anti-angiogenesis, PARP inhibition, and immunotherapy. Unlike existing reviews, we not only evaluate the clinical efficacy of these therapeutic approaches but also explore the mechanisms of drug resistance and the intricate influence of the tumor microenvironment on treatment outcomes. Furthermore, we highlight the application of novel biomarkers, with an emphasis on recent advances in precision medicine. By comparing different therapeutic strategies, this review aims to promote the clinical implementation of personalized treatment, ultimately improving patient prognosis and transforming ovarian cancer into a manageable chronic disease.

## 2 Current research status of targeted therapy for ovarian cancer

**Figure 1:**
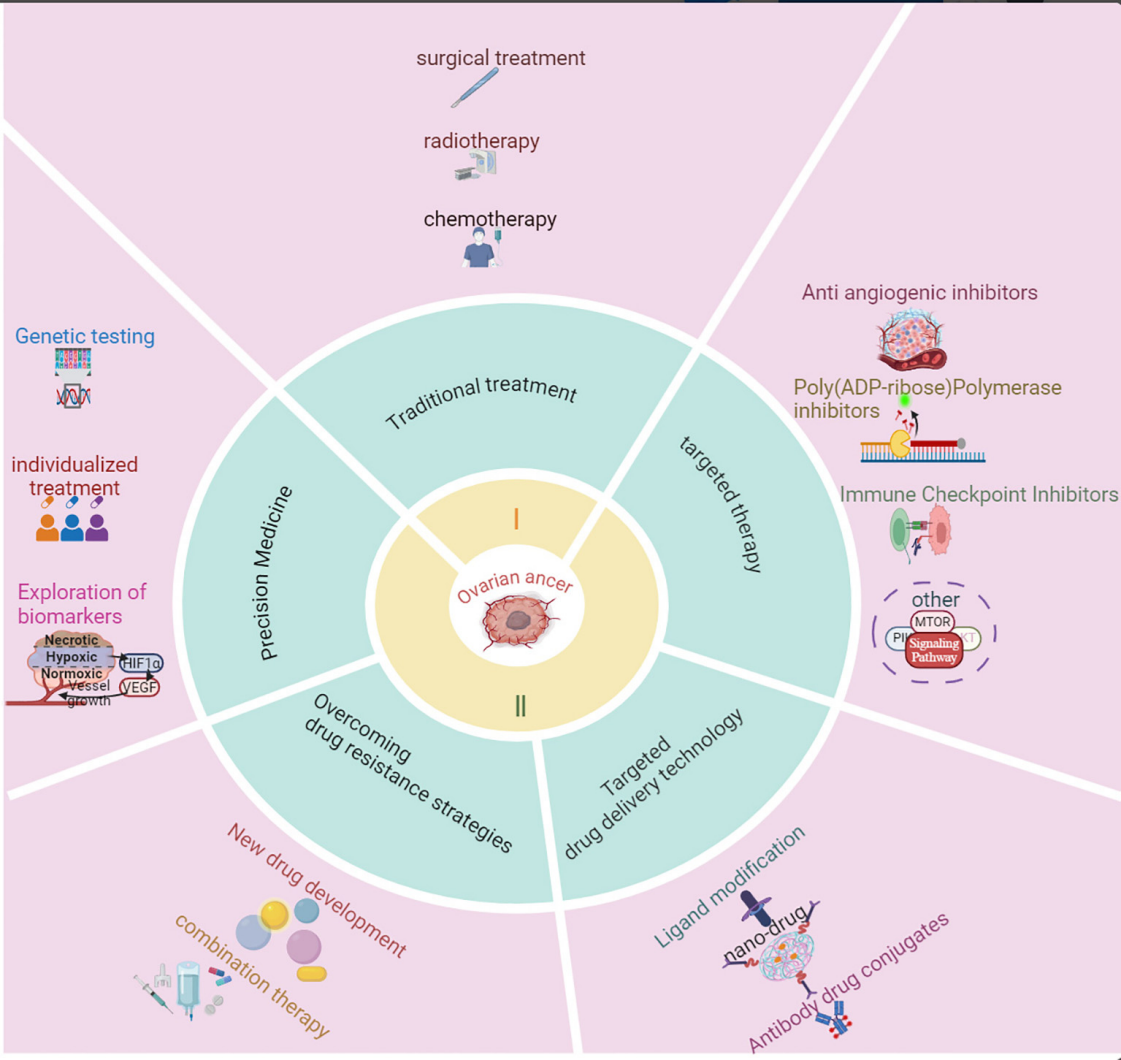
Research progress in the treatment of ovarian cancer. This figure summarizes the key advancements in ovarian cancer treatment, including targeted therapies, immunotherapy, and combination strategies. It highlights the evolution of treatment approaches, from traditional chemotherapy to modern precision medicine, emphasizing breakthroughs such as PARP inhibitors, anti-angiogenic agents, and immune checkpoint inhibitors. The figure also outlines the impact of these innovations on patient outcomes and ongoing challenges in therapeutic resistance and biomarker discovery.

With the rapid advancement of molecular biology and oncology, targeted therapy has become a central focus in ovarian cancer treatment. The paradigm for treating human cancers has shifted from empirical science to evidence-based medicine and, more recently, to targeted therapy. Targeted therapy, which involves controlling tumor growth through specific signaling or metabolic pathways, is a leading area of cancer research (12). Currently, anti-angiogenesis inhibitors (13), PARP inhibitors (PARPis) (14, 15), and ICIs (16) remain central to targeted ovarian cancer therapy. Anti-angiogenesis inhibitors function by blocking the VEGF signaling pathway, preventing the formation of new blood vessels that supply the tumor, thereby limiting tumor growth. Bevacizumab, a monoclonal antibody targeting VEGF, has demonstrated the ability to extend progression-free survival (PFS) and overall survival (OS) in clinical trials and has been approved for ovarian cancer treatment (17, 18). However, due to the emergence of resistance, new treatment strategies are being explored. The use of ICIs in ovarian cancer has also gained attention, as they block the PD1/PD-L1 pathway to restore T-cell anti-tumor activity, enhancing immune system recognition and tumor destruction (19). However, the effectiveness of monotherapy is limited, and current research focuses on combining ICIs with other targeted drugs. Furthermore, PARPis, which target the DNA repair mechanisms in ovarian cancer cells, represent a recent breakthrough, particularly for patients with BRCA1/2 mutations. By inhibiting PARP enzymes involved in DNA repair, particularly in cells with homologous recombination repair deficiency (HRD), PARPis induce tumor cell death through synthetic lethality (20). Olaparib, niraparib, and rucaparib have shown significant efficacy in recurrent ovarian cancer, prolonging PFS and remaining a major area of focus in ovarian cancer research. Other potential targeted therapies are also under investigation, with drugs targeting pathways such as PI3K/AKT/mTOR, EGFR, Notch, and RAS/RAF/MEK/ERK in various stages of clinical development (21).Additionally, novel approaches, such as improving drug delivery methods using ligand-modified nanomedicines, are being explored, Ligands like folate, peptides, hyaluronic acid, and antibodies enhance the targeting of ovarian cancer cells by interacting with specific receptors on their surface (22, 23) (**Figure 1**). While these biopharmaceuticals and emerging therapies have not yet demonstrated the ability to cure ovarian cancer, they hold promise for extending patient survival and potentially transforming ovarian cancer into a manageable chronic disease. With continued progress in these therapies, precision treatment strategies are expected to offer improved outcomes for ovarian cancer patients.

## 3 Anti-angiogenesis drugs

Tumor growth and metastasis rely on the formation of new blood vessels, a process facilitated by the recruitment of endothelial cells and circulating endothelial progenitor cells. Key factors in this process include vascular endothelial growth factor (VEGF), platelet-derived growth factor-BB (PDGF-BB), and fibroblast growth factor-2 (FGF-2) (24). Studies have shown that tumors require neovascularization to sustain growth once their diameter reaches 1 – 3 mm; otherwise, continued expansion becomes challenging. Consequently, inhibiting angiogenesis has emerged as a critical strategy for treating solid tumors (25). Bevacizumab is currently the only anti-angiogenic drug approved for ovarian cancer, making its clinical benefits in this malignancy a focal point of recent research.

**Figure 2:**
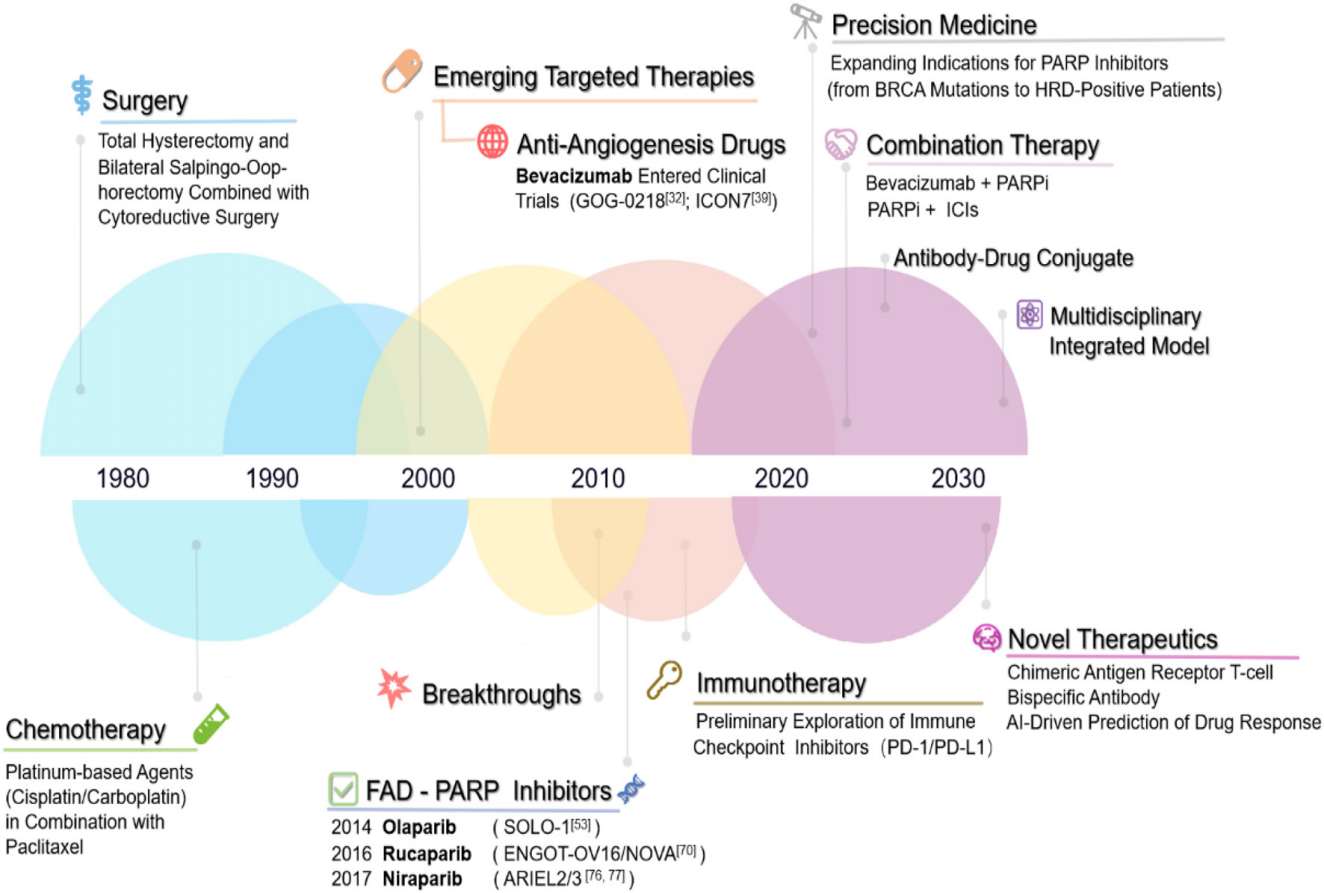
Timeline of major treatment approvals for ovarian cancer. Timeline of Major Treatment Approvals for Ovarian Cancer. 1970s–1990s: Surgical debulking (cytoreductive surgery) and conventional platinumbased chemotherapy regimens were the cornerstone of treatment. 2000s: Emergence of targeted therapies, with preliminary research into anti-angiogenic agents (e.g., bevacizumab) marking the dawn of molecularly driven interventions. 2010s: Landmark breakthroughs in targeted therapy: FDA approval of PARP inhibitors (e.g., olaparib) revolutionized maintenance therapy for BRCA-mutated ovarian cancer, alongside exploratory studies on immune checkpoint inhibitors (e.g., PD-1/PD-L1 blockade). 2020s: Rapid evolution of combinatorial targeted regimens (e.g., PARP inhibitor + anti-angiogenic agent) and integration of personalized precision medicine through genomic profiling and biomarker-guided strategies. Future Directions: AI-driven diagnostics and next-generation gene-editing therapies are anticipated to redefine therapeutic paradigms.

Vascular endothelial growth factor (VEGF) is a key pro-angiogenic factor in ovarian cancer, and its overexpression is associated with poor prognosis (26). Bevacizumab, a monoclonal antibody targeting VEGF-A, has been the primary anti-angiogenesis agent used in ovarian cancer (27–29). It was first approved in 2014 for use in combination with non-platinum chemotherapy in the treatment of platinum-resistant recurrent ovarian cancer (30) (**Figure 2**: Timeline of Major Treatment Approvals for Ovarian Cancer). A clinical trial extending bevacizumab treatment to 30 months did not improve progression-free survival (PFS) or overall survival (OS) in ovarian cancer patients (31). Consequently, the standard treatment protocol remains cytoreductive surgery followed by 15 months of bevacizumab therapy (32). The phase III ICON7 clinical trial demonstrated that the progression-free survival (PFS) in the bevacizumab group was 19.0 months, showing a slight improvement compared to 17.3 months in the standard treatment group. Notably, at the 36-month follow-up, high-risk patients experienced a more significant PFS benefit. Additionally, overall survival (OS) was significantly prolonged in the high-risk group (39.3 months vs. 34.5 months), whereas no apparent difference was observed in non-high-risk patients. An interesting phenomenon was identified in this study: the risk of early disease progression was lower in the bevacizumab-treated group than in the control group; however, it gradually increased from approximately six months onward and reached the same level as the control group by the time treatment was discontinued at around 12 months (18). Additionally, when patients were categorized by serous and non-serous subtypes, a rebound effect was only observed in the serous subtype. Furthermore, the GOG-0218 study further confirmed that bevacizumab can improve the prognosis of patients with poor chemotherapy sensitivity, extending progression-free survival (PFS) by approximately four months in patients with advanced epithelial ovarian cancer (33). This trial confirmed that patients with poor chemotherapy responses derive significant benefit from continued bevacizumab therapy. Bevacizumab is especially effective in high-risk, chemotherapy-resistant patients, improving their PFS/OS. However, in BRCA1/2 mutation-positive patients, continued bevacizumab treatment did not show survival benefits, and transition to olaparib therapy is recommended (34). Data from ICON-7 also revealed that in the non-high-risk group, there was no significant difference in overall survival between the bevacizumab and standard chemotherapy groups, but in the poor prognosis group, there was a significant survival benefit with bevacizumab (34.5 months vs. 39.3 months) (35). A study involving 406 patients treated with carboplatinbased chemotherapy found a significant difference in PFS, with the bevacizumab group having a median PFS of 11.8 months versus 8.8 months in the chemotherapy group (36). Additionally, phase II clinical studies have demonstrated that the combination of bevacizumab and immune checkpoint inhibitors (ICIs) exhibits promising antitumor activity in ovarian cancer patients, with manageable toxicity (37–39).

In conclusion, bevacizumab, as a monoclonal antibody targeting VEGF-A, has demonstrated significant clinical benefits in ovarian cancer treatment, particularly for high-risk and chemotherapy-resistant patients. While its long-term efficacy remains limited, further studies are necessary to explore the mechanisms of resistance to bevacizumab, including the activation of alternative angiogenic pathways and their impact on tumor immune evasion.

## 4 Immune checkpoint inhibitors

Research on immune checkpoint inhibitors (ICIs) in ovarian cancer has been progressing rapidly. Tumor-infiltrating T cells serve as indicators of the host’s immune response to tumor antigens, and their presence correlates positively with patient progression-free survival (PFS) and overall survival (OS) (40). ICIs enhance the activity of cytotoxic immune cells, promoting the destruction of tumor cells (41).

Pembrolizumab, an anti-PD-1 antibody, primarily binds to PD-L1 on T cells within tumor cells, preventing the interaction between PD-1 and its ligand, PD-L1. This action helps maintain T-cell activity, leading to tumor cell apoptosis and subsequent elimination (42). Given the strong antitumor activity of PD-1 inhibitors in other cancers, such as lung cancer, melanoma, and renal cancer, Pembrolizumab has been actively investigated in ovarian cancer in recent years (43). A Phase II clinical study was initially conducted in 20 platinumresistant ovarian cancer patients, divided into two groups (10 patients each) and treated with nivolumab (1 mg/kg and 3 mg/kg intravenous injection). The overall response rate (ORR) was 15%, with two patients in the 3 mg/kg group achieving a complete response (CR) (44). Notably, patients in this study were not selected based on their PD-1 status. Subsequently, a Phase Ib clinical trial was conducted using Nivolumab in 26 PD-L1positive advanced metastatic ovarian cancer patients, yielding similar results (45); however, this study lacked a control group. Two years later, a larger-scale Phase II clinical trial was conducted, Patients with advanced PD-L1-positive ovarian cancer were stratified, and their objective response rate (ORR) was evaluated based on the combined positive score (CPS) of PD-L1 expression (46). The results indicated that higher PD-L1 expression levels were associated with a greater ORR to pembrolizumab across all patients. Specifically, patients with CPS ≥10 demonstrated the most significant benefit, with an ORR of 17.1%, whereas those with CPS <1 had an ORR of only 5.0%, suggesting that patients with high PD-L1 expression exhibit a better response to pembrolizumab treatment (46). Furthermore, despite variations in prior chemotherapy exposure (1–3 lines vs. 4–6 lines), the ORR did not significantly decline with an increasing number of previous treatment lines, indicating that the efficacy of pembrolizumab is not substantially affected by prior therapeutic regimens (46) In conclusion, Although pembrolizumab has shown some clinical benefits in ovarian cancer, its overall therapeutic efficacy remains limited. Immunotherapy primarily enhances antitumor immune responses by activating the patient’s immune system; however, the immune evasion mechanisms of ovarian cancer contribute to its poor responsiveness to such treatments (47). Studies have shown that the immune microenvironment of ovarian cancer is highly complex, with tumor-infiltrating immune cells exhibiting functional suppression (48). This suppression weakens the antitumor activity of T cells, thereby compromising the effectiveness of immunotherapy and resulting in a lower response rate in ovarian cancer compared to other malignancies (49).Secondly, the efficacy of PD-1/PDL1 inhibitors as monotherapy remains controversial. Some studies suggest that PD-L1 alone is not a fully reliable biomarker for predicting immunotherapy response. Although patients with PD-L1-positive expression tend to respond better to pembrolizumab, a considerable proportion of PD-L1-positive patients fail to derive significant clinical benefits (50). Moreover, some PD-L1-negative patients also exhibit favorable responses to immunotherapy, highlighting the limitations of PD-L1 as a sole predictive biomarker (51). In fact, the complexity of immune responses and the heterogeneity of the tumor immune microenvironment suggest that PD-L1 expression alone is insufficient to fully predict therapeutic outcomes.

## 5 PARP inhibitors

PARP enzymes are activated in response to DNA damage and play a critical role in repairing single-strand DNA breaks. These enzymes help cells repair damage, maintain genomic stability, and influence gene transcription and expression through nuclear protein modifications. Currently approved PARP inhibitors for ovarian cancer, such as olaparib, niraparib, and rucaparib, have demonstrated significant clinical efficacy, particularly in patients with BRCA1/2 mutations, where they effectively induce tumor cell death through the synthetic lethality mechanism (52, 53). However, the clinical performance of different PARP inhibitors varies considerably, which is not only related to the intrinsic properties of the drugs but also closely linked to the patient’s genetic background and treatment regimen. As the first approved PARP inhibitor, olaparib has been validated in multiple clinical trials for its efficacy in patients with BRCA mutations and homologous recombination deficiency (HRD). Despite its favorable therapeutic effect in platinum-sensitive recurrent ovarian cancer, olaparib faces challenges related to resistance during long-term maintenance therapy, particularly in patients without BRCA mutations (54). In contrast, niraparib has shown more pronounced clinical benefits, even in patients without BRCA mutations, as it extends progression-free survival (PFS) (55). However, its use is limited in certain patient populations due to notable adverse effects, such as thrombocytopenia (56). Rucaparib, on the other hand, exhibits a stronger ability to overcome resistance among PARP inhibitors, particularly in BRCA wild-type patients, making it a promising therapeutic option.

Moreover, studies have shown that beyond BRCA mutations, many sporadic ovarian cancers also exhibit other DNA repair deficiencies, such as homologous recombination deficiency (HRD), rendering these patients partially sensitive to PARP inhibitors (PARPi) (57).The HRD scoring system has become a crucial tool in clinical practice for identifying HRD-positive ovarian cancer patients. HRD scoring is typically based on several key indicators, including BRCA1/2 mutations, loss of heterozygosity (LOH), and tumor mutational burden (TMB) (58). By comprehensively evaluating these parameters, HRD scoring enables a more accurate identification of patients with HRD, thereby providing them with the opportunity to receive PARP inhibitors or other targeted therapies. Furthermore, the HRD scoring system effectively distinguishes prognostic differences between BRCA-mutated and non-mutated patients and predicts their sensitivity to PARP inhibitors (59).

### 5.1 Olaparib

Olaparib is an oral PARP inhibitor (PARPi) that has demonstrated robust antitumor activity in patients with metastatic ovarian cancer harboring germline BRCA mutations (60). It is the most extensively studied PARPi in ovarian cancer. In 2009, the synthetic lethal interaction between PARPi and BRCA1/BRCA2 mutations was first confirmed (61). Subsequently, a multicenter, double-blind, randomized Phase II clinical trial involving 265 patients with high-grade serous ovarian cancer and BRCA1/2 germline mutations revealed that progression-free survival (PFS) was significantly longer in the Olaparib group (8.4 months) compared to the placebo group (4.8 months) (62). A large-scale Phase III study, SOLO1, further demonstrated that among 196 patients with BRCA1/2 mutations and platinum-sensitive recurrent ovarian cancer, Olaparib treatment resulted in a PFS of 19.1 months, compared to just 5.5 months in the placebo group. The Olaparib group exhibited significantly prolonged PFS with manageable adverse effects (63). Based on these clinical trial results, the U.S. Food and Drug Administration (FDA) approved Olaparib tablets (Lynparza, AstraZeneca) for maintenance treatment of adults with recurrent epithelial ovarian cancer, fallopian tube cancer, or primary peritoneal cancer who had achieved a complete or partial response to platinum-based chemotherapy. Recent follow-up data from the SOLO1 study continued to show clinical benefits, with a median PFS extending beyond 4.5 years, even after treatment discontinuation (64). Furthermore, 7-year follow-up results published in 2023 indicated significant and durable clinical benefits with adverse events primarily of grade 1-2 severity (65) (66). The SOLO2 final analysis also demonstrated that Olaparib maintenance therapy in platinum-sensitive recurrent ovarian cancer with BRCA mutations extended overall survival by 12.9 months (67). However, recent analyses by J. S. Frenel et al. suggested that re-treatment with platinum-based chemotherapy following prior Olaparib therapy may be less effective compared to patients who had not received Olaparib (68). Despite this, Olaparib maintenance therapy has shown efficacy across all age groups in platinum-sensitive recurrent ovarian cancer patients with BRCA mutations (69). Moreover, in a Phase II trial comparing Olaparib with pegylated liposomal doxorubicin (PLD) in platinum-resistant or partially platinum-sensitive recurrent ovarian cancer patients with BRCA mutations, no significant differences in PFS/OS were observed (70) (71),However, in a cohort of 31 platinum-resistant ovarian cancer patients, irrespective of BRCA status, combination therapy showed significant activity, prompting further investigation (72). Some studies suggest that Olaparib can improve PFS even in patients without BRCA mutations or homologous recombination deficiency (HRD) in first-line and platinum-sensitive recurrent ovarian cancer treatment (73). Molecular analyses by Takahiro Nozaki et al. revealed that ovarian cancer patients with homologous recombination repair-related gene mutations (HRRm) had longer PFS compared to those without HRRm, suggesting that HRRm could serve as a predictive biomarker for PARPi efficacy (74). Furthermore, the L-MOCA trial analysis identified HRD as an effective biomarker for platinum-sensitive recurrent ovarian cancer, while high PD-L1 expression in BRCAmutated patients appeared to reduce Olaparib efficacy (75). This finding provides a rationale for combination therapies.

In conclusion, Olaparib remains a standard first- or second-line treatment for platinum sensitive recurrent ovarian cancer. However, unresolved issues include the mechanisms of resistance, the need for better predictive biomarkers, and the development of unified strategies for managing drug toxicities. Addressing these challenges will optimize the therapeutic benefits of Olaparib for ovarian cancer patients.

### 5.2 Niraparib

Niraparib demonstrates significant antitumor activity in metastatic ovarian cancer and has shown favorable clinical efficacy in ovarian cancer patients (76). The earliest phase I dose escalation study established the optimal dose of niraparib as 300 mg once daily, with pharmacodynamic analysis indicating that doses of ≥60 mg/day could elicit antitumor activity, while PARP inhibition exceeded 50% at doses of ≥80 mg/day (77). In the subsequent phase III randomized, double-blind clinical trial (ENGOT-OV16/NOVA), researchers stratified 553 ovarian cancer patients based on BRCA mutation status into the gBRCA group (n = 203) and the non-gBRCA group (n = 350). The median progression-free survival (PFS) was significantly longer in the niraparib group compared to the placebo group in both cohorts (gBRCA group: 21.0 vs. 5.5 months; non-gBRCA group: 12.9 vs. 3.8 months), demonstrating that niraparib significantly prolonged PFS regardless of BRCA or homologous recombination deficiency (HRD) status (78). This conclusion was further validated in a large-scale clinical trial involving 733 patients (79). Based on these data, the United States and Europe approved niraparib for first-line maintenance therapy in patients with platinum-sensitive recurrent ovarian cancer. Long-term follow-up data indicated that niraparib maintained a stable safety profile and patient quality of life (QOL). A three-year follow-up analysis of the ENGOT-OV16/NOVA trial showed that niraparib did not significantly reduce QOL, with the primary grade 3–4 adverse events being manageable hematologic toxicities (80). The 3.5-year follow-up of the PRIMA/ENGOT-OV26/GOG3012 trial further confirmed that niraparib significantly improved PFS (four-year progression-free survival rate: 24% vs. 14%), with toxicity not worsening over time (81). The 2024 overall survival (OS) analysis revealed that the five-year survival rate in the niraparib group was twice that of the placebo group, further substantiating its long-term survival benefits (82).

In summary, niraparib, as an oral PARP inhibitor, demonstrates significant efficacy in the maintenance treatment of ovarian cancer. It effectively prolongs progression-free survival, regardless of the presence of BRCA mutations or HRD status. Numerous clinical trials have confirmed its antitumor activity, and the drug’s side effects are relatively manageable, without significantly impacting patients’ quality of life. Thus, niraparib has become a standard maintenance therapy for platinum-sensitive recurrent ovarian cancer. Long-term follow-up has further solidified its importance in ovarian cancer treatment. However, optimizing dosage and treatment regimens for patients with different genotypes to improve efficacy and reduce side effects remains a key area for future research. Furthermore, while niraparib shows efficacy in both BRCA mutation-positive and HRD-unknown patients, additional research into biomarkers could help identify patient subgroups more likely to benefit from PARP inhibitor therapy. As our understanding of the molecular mechanisms of ovarian cancer advances, the indications for niraparib may expand, and the exploration of personalized treatment strategies will provide more precise therapeutic options for patients.

### 5.3 Rucaparib

Rucaparib is an effective PARP inhibitor targeting PARP-1, PARP-2, and PARP-3. A phase I dose-escalation study (n = 78) evaluated the efficacy of rucaparib in BRCA-mutated ovarian cancer, with patients receiving intravenous or oral administration (240–840 mg, twice daily). The study established 600 mg BID as the optimal dose and observed significant clinical benefits in BRCA-mutated patients (83). Additionally, 79% of the drug was excreted via feces (84). In another phase I study (n = 42), all patients received rucaparib 600 mg BID, with BRCA1/2-mutated patients demonstrating an objective response rate (ORR) of 59.5% based on RECIST criteria, and an ORR of 83.3% when assessed using RECIST/GCIG CA-125 criteria (85). The phase II ARIEL2 study (n = 192) was the first to indicate that loss of heterozygosity (LOH) could predict rucaparib efficacy. The study found that PFS in BRCA-mutated patients (12.8 months) was significantly longer than in the LOH-high (5.7 months) and LOH-low (5.2 months) groups, suggesting that rucaparib could be extended for use in BRCA wild-type patients (86). Based on these findings, the FDA granted accelerated approval for rucaparib in December 2016 for advanced BRCA-mutated ovarian cancer in patients who had received ≥2 prior lines of chemotherapy. A subsequent phase III study (n = 564) further assessed the role of rucaparib in the maintenance treatment of platinum-sensitive, high-grade serous or endometrioid ovarian cancer. Patients were stratified by BRCA mutation and homologous recombination deficiency (HRD) status and randomized to receive rucaparib 600 mg BID or placebo. The results demonstrated a significant PFS benefit with rucaparib compared to placebo, including in BRCA-mutated patients (16.6 vs. 5.4 months), HRD-positive patients (13.6 vs. 5.4 months), and the overall intent-to-treat population (10.8 vs. 5.4 months) (87). Given the clinical benefits demonstrated in this study, both the FDA and EMA approved rucaparib for the maintenance treatment of high-grade serous ovarian cancer (HGOC), regardless of BRCA1/2 status.

**Figure 3:**
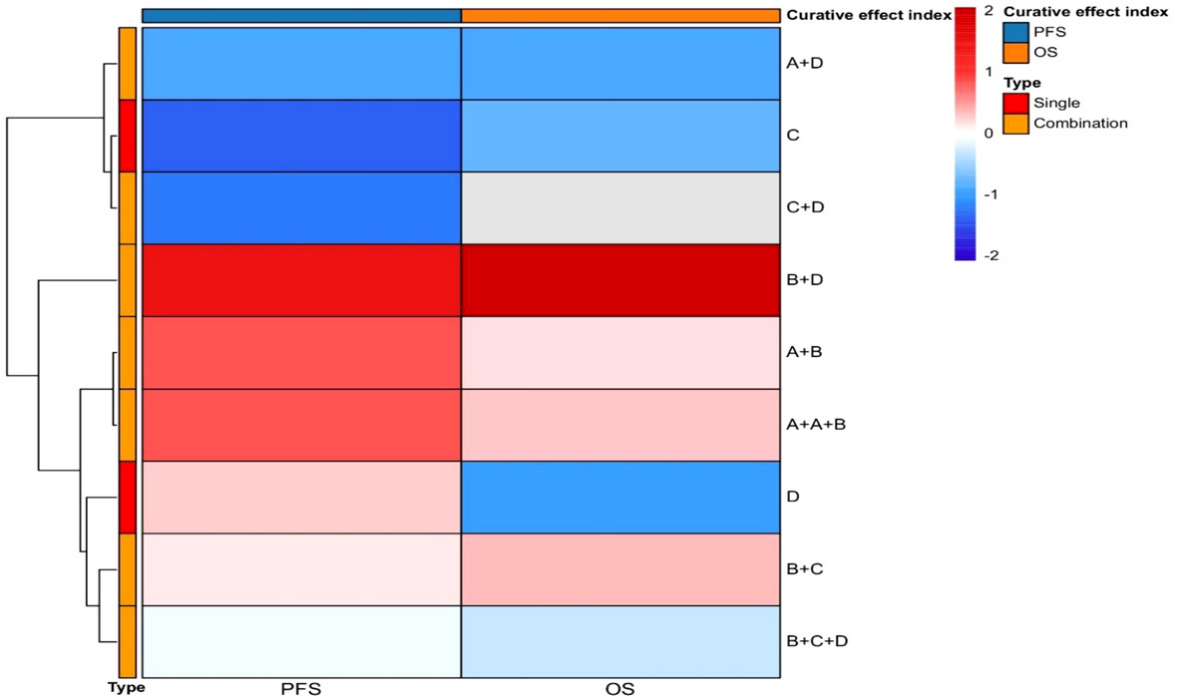
Efficacy of targeted therapies in ovarian cancer. The x-axis delineates efficacy endpoints, including progression-free survival (PFS) and overall survival (OS). The y-axis enumerates therapeutic agents evaluated in clinical trials, comprising both monotherapy and combination regimens. Symbolic designations are standardized as: A (chemotherapeutic agents), B (anti-angiogenic drugs), C (immune checkpoint inhibitors), D [poly(ADP-ribose) polymerase (PARP) inhibitors], with plus signs (+) indicating drug combinations. This heatmap systematically encapsulates the therapeutic efficacy profiles of both single-agent and combinatorial approaches that have defined the evolutionary landscape of targeted therapies in ovarian cancer.

In summary, rucaparib, as an effective PARP inhibitor, demonstrates significant efficacy in treating advanced ovarian cancer patients with BRCA mutations. Clinical studies have shown that both BRCA mutation carriers and patients with homologous recombination deficiency (HRD) benefit from rucaparib, with particularly prolonged PFS observed in the BRCA mutation group. Moreover, LOH status-based assessments help identify subgroups of BRCA wild-type patients who may benefit from rucaparib therapy, thus broadening the potential applications of PARP inhibitors. Following the positive results from Phase III clinical studies, rucaparib has been approved by the FDA and EMA for maintenance treatment of HGOC, irrespective of BRCA1/2 status. This approval represents a significant advance in ovarian cancer treatment strategies, providing patients with more therapeutic options. Future research may explore the application of rucaparib in other types of cancer and focus on optimizing dosage and treatment regimens to enhance efficacy and minimize side effects. Furthermore, in-depth studies on HRD biomarkers could help more accurately identify patients who are likely to benefit from PARP inhibitor therapy (**Figure 3**: Efficacy of Targeted Therapies in Ovarian Cancer).

## 6 Combination of targeted drugs

### 6.1 Combination of PARP inhibitors with signal pathway inhibitors

Signal pathway inhibitors have garnered significant attention in ovarian cancer research, particularly those targeting the PI3K/AKT/mTOR pathway. This pathway is frequently activated in various cancers, especially ovarian cancer, and plays a crucial role in biological processes such as cell proliferation, survival, and metabolism. Consequently, targeting the PI3K/AKT/mTOR pathway has become a promising therapeutic strategy in ovarian cancer. In 2019, Meran Keshawa Ediriweera and colleagues published a systematic review on single-agent therapies targeting this pathway (88), which will not be further discussed here. This section will focus on research regarding the combination of PI3K inhibitors and PARP inhibitors in ovarian cancer treatment. While research on this combination strategy in ovarian cancer remains limited, its promising efficacy in breast cancer offers hope for its applicability to ovarian cancer as well (89–91). Currently, studies are still in the early stages (Phase I/Ib).In a preclinical study, Dong Wang and colleagues demonstrated through *in vitro* experiments that combining PI3K inhibitors with PARP inhibitors enhances sensitivity to PARP inhibition by inhibiting DNA homologous recombination repair mechanisms. This combination showed potential therapeutic benefits, particularly in ovarian cancer cells with PIK3 mutations or wild-type PIK3 (92). This finding provides a theoretical basis for subsequent clinical studies and highlights the potential to extend this therapy to a broader range of ovarian cancer patients, especially those who do not respond to single-agent PARP inhibitor therapy. The first Phase I clinical trial, conducted in 2012, enrolled 118 patients with recurrent triple-negative breast cancer or high-grade serous ovarian cancer to evaluate the safety and tolerability of combining PI3K inhibitors with PARP inhibitors (93). Another Phase I trial assessed the clinical benefits of combining Olaparib with alpelisib (a PI3K-α inhibitor) in recurrent platinum-resistant ovarian cancer, showing an objective response rate (ORR) of 36% (94), similar to the results from combining Olaparib with BKM120 (another PI3K-α inhibitor) (95). In an evaluation of 49 ovarian cancer patients, the combination of Olaparib and the PI3K inhibitor vistusertib resulted in an ORR of 20%, with manageable adverse effects (96).

In summary, the PI3K/AKT/mTOR pathway is a critical target in ovarian cancer therapy, and the combination of PI3K inhibitors with PARP inhibitors shows promise in some ovarian cancer patients. Research on single-agent therapies is more advanced, but existing evidence suggests that combination therapy may help overcome resistance to single-agent PARP inhibitor therapy. Basic studies and Phase I clinical trials have confirmed that combining PI3K and PARP inhibitors enhances drug sensitivity by inhibiting DNA homologous recombination repair mechanisms in ovarian cancer cells with PIK3 mutations or wild-type PIK3. This provides an important basis for further clinical research, and preliminary data suggest promising efficacy. However, most current studies remain in Phase I/Ib, and further validation of efficacy and safety is required. Although combination therapy has shown success in recurrent platinum-resistant ovarian cancer, ORR varies across studies (20%–36%). Future research should focus on optimizing dosing regimens, reducing side effects, and identifying biomarkers to select the most responsive patient populations.

### 6.2 Combination of PARP inhibitors with anti-angiogenesis inhibitors

Both anti-angiogenesis drugs and PARP inhibitors have demonstrated activity as single agents in recurrent ovarian cancer (97). Mechanistically, combining anti-angiogenesis therapy with PARP inhibitors may enhance anti-tumor activity (98). Studies have shown that anti-angiogenesis drugs influence homologous recombination repair (HRR) through several mechanisms, such as inhibiting angiogenesis, inducing tumor hypoxia, and downregulating key HRR factors like BRCA1/2 and RAD51 (99). Consequently, the combination of these two classes of drugs has garnered significant attention. In recent years, clinical research on the combination of bevacizumab (an anti-angiogenesis drug) and PARP inhibitors has expanded. A study involving 105 ovarian cancer patients found that 76% of patients with homologous recombination deficiency (HRd) treated with bevacizumab and PARP inhibitors had a progression-free survival (PFS) of 18 months, significantly higher than the 47% observed in the HRd-negative group. After a median follow-up of 28.7 months, the PFS for the HRd-positive group was 28.3 months, compared to 12.1 months for the negative group. Adverse reactions were consistent with those of single-agent therapy, predominantly grade 1–2 (100). Another Phase II trial with 48 patients demonstrated that the combination of Niraparib (a PARP inhibitor) and bevacizumab significantly improved PFS (11.9 months vs. 5.5 months) compared to Niraparib alone (101). In a recent clinical trial, 469 ovarian cancer patients were treated with the same regimen. Among 138 patients with BRCA mutations, the combination of bevacizumab and PARP inhibitors showed a longer PFS (36.4 months vs. 18.6 months) compared to the control group. The most common adverse event was hypertension (102). In a subsequent Phase III trial, 806 BRCA-mutated ovarian cancer patients received the combination therapy, resulting in a 5-year PFS rate of 35% and an overall survival (OS) rate of 70%, compared to 28% and 31%, respectively, in the bevacizumab-only group (103). In a further Phase III trial (NCT02477644), 535 out of 806 patients received Olaparib combined with bevacizumab. After a median follow-up of 22.9 months, the PFS for the combination group was significantly longer (22.1 months vs. 16.1 months) compared to the placebo group. Notably, patients with HRD-positive tumors had a more significant difference in PFS (37.2 vs. 17.7 months) (104), and a higher percentage of patients in the combination group had no disease progression after 5 years (46.1% vs. 19.2%). However, patients with BRCA-negative tumors did not benefit from this therapy (32).

In conclusion, recent studies have shown that combining anti-angiogenesis drugs with PARP inhibitors offers long-term clinical benefits. BRCA mutations can serve as a biomarker for the use of Olaparib and bevacizumab as maintenance therapy. However, efficacy is lower in BRCA-negative and HRp subgroups, suggesting that future research should focus on developing new biomarkers or combination strategies for these subgroups. Additionally, while current studies mainly focus on extending PFS, future research should aim to improve overall survival (OS) and quality of life for patients. Although hypertension is the most common adverse event, a standardized approach to managing side effects has not yet been established. Future studies should explore strategies to address side effects and improve patient quality of life. Furthermore, predictive therapeutic biomarkers are scarce, though a clinical trial has suggested that visceral fat density (VFD) may predict response to bevacizumab treatment. High VFD patients may benefit more from initial bevacizumab therapy (105). Angiopoietin-2 (Ang-2) has been identified as a potential biomarker for ovarian cancer spread to lymph nodes and is associated with patient prognosis, potentially serving as a marker to identify patients who would benefit from bevacizumab treatment (106). Additionally, fibroblast growth factor receptors and their ligands (FGFRs/FGFs) have independent prognostic value (107). Future research should focus on discovering new biomarkers to help more ovarian cancer patients benefit from this therapy.

### 6.3 Combination of PARP inhibitors with immune checkpoint inhibitors

**Figure 4:**
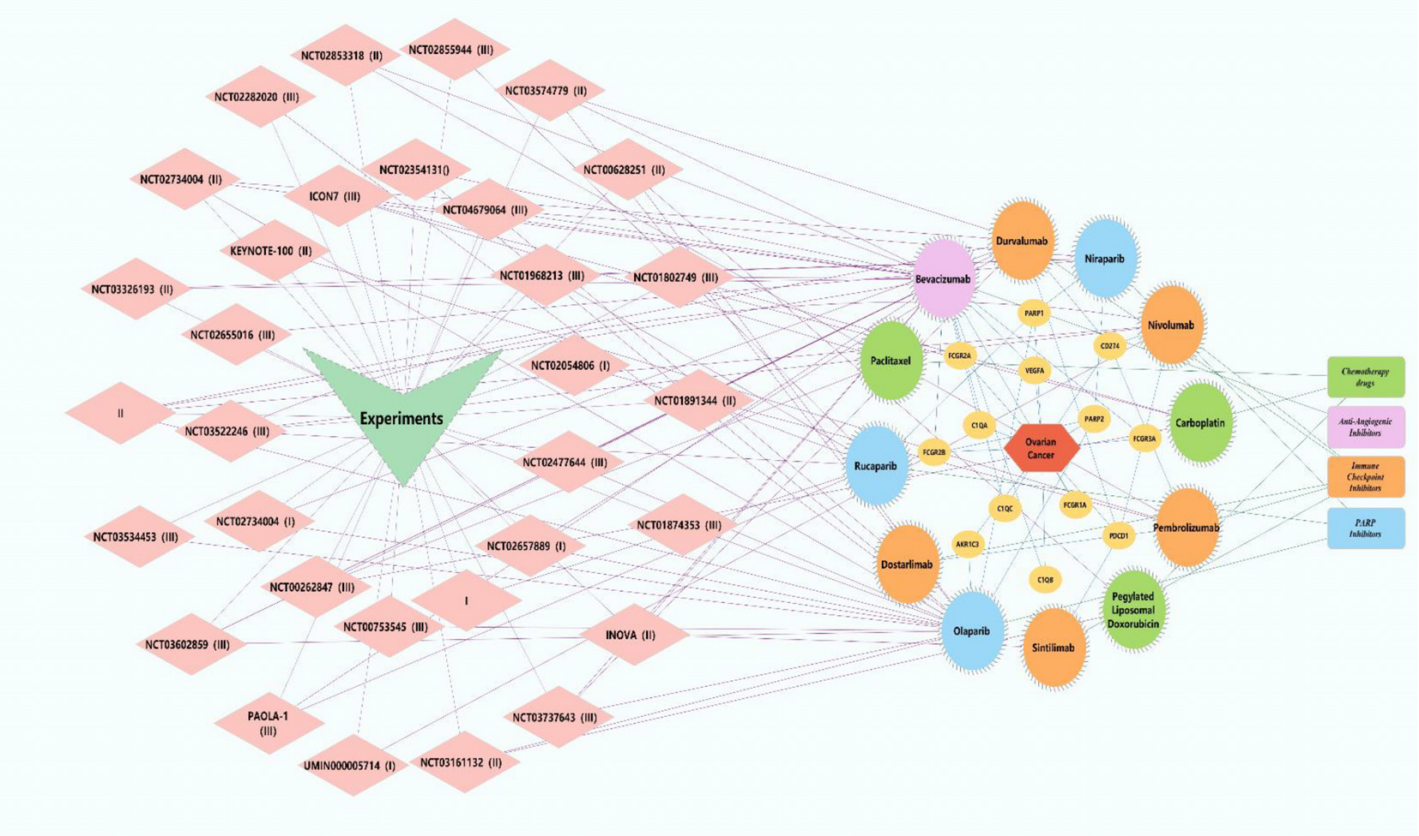
Key trials and combination therapies of targeted therapies approved for ovarian cancer. Pink diamonds represent clinical trials associated with targeted therapies for ovarian cancer. Yellow circles denote investigational drugs and overlapping genes linked to ovarian cancer. Green rectangles symbolize chemotherapy agents, with green circles indicating chemotherapy drugs utilized in the trials. Purple rectangles represent antiangiogenic drugs, and purple circles correspond to anti-angiogenic agents employed in the trials. Orange rectangles signify immune checkpoint inhibitors, while orange circles denote immune checkpoint inhibitors used in the trials. Blue rectangles represent poly(ADP-ribose) polymerase (PARP) inhibitors, and blue circles indicate PARP inhibitors administered in the trials. Green V-shapes and red hexagons represent clinical trials and ovarian cancer, respectively.

The combination of PARP inhibitors (PARPis) and immune checkpoint inhibitors has shown significant anti-tumor activity in various advanced solid tumors (46–109). In ovarian cancer, PARPis have been shown to enhance the effects of immune checkpoint blockade (110), promoting tumor T-cell activation (111), which opens new therapeutic avenues for advanced ovarian cancer. Studies suggest that DNA damage induced by PARPis, coupled with an increased mutational burden, may lead to the generation of more neoantigens. These neoantigens can be more effectively recognized by the immune system, potentially resulting in stronger anti-tumor immune responses, improved treatment outcomes, and enhanced patient survival, especially when combined with immune checkpoint inhibitors (112, 113).The earliest Phase I dose-escalation study (NCT02484404) of PARPi combined with immune checkpoint inhibitors included 26 patients with advanced ovarian cancer, 12 of whom received the combination therapy of durvalumab and olaparib. The regimen involved 300 mg olaparib and 1,500 mg durvalumab, administered intravenously every four weeks. The objective response rate (ORR) was 17%, and the disease control rate (DCR) was 83%. The most common adverse event (AE) observed was hematologic toxicity (114). In another Phase I clinical trial involving 60 patients with platinum-resistant, platinum-refractory, and platinum-sensitive ovarian cancer, the effective dose for the combination therapy was determined to be 200 mg daily of niraparib and 200 mg of pembrolizumab, administered intravenously every 21 days (115).A Phase II study enrolling 35 ovarian cancer patients who had received at least one prior treatment primarily assessed the efficacy and safety of durvalumab combined with olaparib. Although the study did not achieve its pre-specified ORR target (14%), the DCR was as high as 71%, with 34% of patients experiencing clinical benefit t (116). In another Phase II trial involving 32 patients with BRCA1/2 mutations and platinum-sensitive ovarian cancer, the combination therapy achieved a 12-week DCR of 81% and an ORR of 63%. Among patients who had undergone one to two prior chemotherapy regimens, the ORR was even higher, reaching 68% (117). Additionally, a study of 41 patients with BRCA mutations and homologous recombination (HR) deficiencies demonstrated promising anti-tumor activity with a combination of a BRCA inhibitor, immune checkpoint inhibitors, and bevacizumab (ORR of 17.1%, DCR of 73.2%). However, all patients experienced Grade 3 or higher adverse events, leading to the discontinuation of one or more drugs (118). Interestingly, this triplet regimen showed durable efficacy in non-BRCA-positive patients (ORR of 87.1%), with a safety profile comparable to that of the olaparib and durvalumab combination (119). However, due to the small sample size, the study lacked randomization and a control group, and thus its results must be interpreted with caution. Despite these limitations, the study demonstrated significant clinical benefits, leading to the initiation of several large Phase III trials to validate these findings. For example, the global, multicenter, randomized, double-blind, controlled Phase III trial (NCT03602859) will enroll 1,402 patients to compare the efficacy of dostarlimab combined with standard chemotherapy with or without bevacizumab, versus niraparib maintenance therapy following chemotherapy. The primary endpoint is progression-free survival (PFS), and the results are expected by June 2026 (120). Another Phase III trial will compare standard platinum-based chemotherapy (e.g., carboplatin or cisplatin) combined with bevacizumab maintenance therapy to a regimen including durvalumab, or a triplet regimen with durvalumab, bevacizumab, and PARPi (e.g., olaparib). This trial is expected to release data by March 2028 (121). A third Phase III trial with 427 patients with recurrent ovarian cancer, fallopian tube cancer, or primary peritoneal cancer will compare niraparib combined with dostarlimab versus chemotherapy alone, with results expected in January 2025 (122). Finally, a Phase III trial involving 1,000 newly diagnosed ovarian cancer patients will evaluate rucaparib and nivolumab as maintenance therapy following first-line treatment, with results expected by 2030 (123)(**Figure 4** Key Trials and Combination Therapies of Targeted Therapies Approved for Ovarian Cancer). In recent years, the combination of PARP inhibitors and immune checkpoint inhibitors has demonstrated promising anti-tumor activity, particularly in patients with advanced or recurrent ovarian cancer. Although Phase III clinical trial data have not yet been released, the clear clinical benefits observed in earlier Phase I and II trials—whether combining PARPi with immune checkpoint inhibitors or anti-angiogenesis inhibitors—suggest that these approaches may yield positive outcomes. In the coming years, focus should shift to the results of these pivotal clinical trials, which could provide more precise and personalized treatment options for ovarian cancer, improving both progression-free survival (PFS) and overall survival (OS). Additionally, future research should aim to identify effective biomarkers for patient selection, ensuring that therapies benefit those most likely to respond while minimizing unnecessary treatments. Moreover, safety profiles, particularly regarding adverse events, should be closely monitored, and management strategies should be developed to prevent drug discontinuation and improve patient quality of life.

## 7 Other targeted therapeutic strategies

In addition to PARP inhibitors, immune checkpoint inhibitors, and anti-angiogenic agents, other targeted therapeutic strategies, such as tyrosine kinase inhibitors (TKIs), lipid metabolism-targeting drugs, gene therapy, and cell cycle checkpoint inhibitors, are currently in clinical trial stages. tyrosine kinase inhibitors (TKIs):TKIs represent a crucial class of targeted agents for ovarian cancer treatment. By inhibiting tyrosine kinase signaling pathways within tumor cells, TKIs effectively suppress tumor cell proliferation, metastasis, and angiogenesis (124). Several tyrosine kinase inhibitors are under investigation and have been applied clinically in ovarian cancer, particularly those targeting vascular endothelial growth factor receptor (VEGFR) and epidermal growth factor receptor (EGFR). TKIs targeting VEGFR, such as sorafenib, sunitinib, and lenvatinib, have been explored in clinical trials for ovarian cancer, primarily aiming to inhibit tumor angiogenesis (125). Additionally, TKIs targeting EGFR, such as erlotinib and gefitinib, are being evaluated in clinical trials for ovarian cancer, showing potential therapeutic efficacy particularly in ovarian cancer subtypes with EGFR overexpression or mutations (126). Lipid Metabolism-Targeted Therapeutic Agents:Lipid metabolism plays a critical role in ovarian cancer progression, drug resistance, and immune regulation, making it an emerging research focus. Fatty acid synthase (FASN) is overexpressed in ovarian cancer and promotes tumor growth. FASN inhibitors, such as TVB-2640 (currently in phase I clinical trials) (127)and orlistat (an FDA-approved drug), have been found to effectively suppress tumor cell proliferation (128). Furthermore, abnormal cholesterol metabolism influences tumor cell survival and drug resistance. Atorvastatin reduces cholesterol levels by inhibiting HMG-CoA reductase, while liver X receptor (LXR) agonists suppress tumor growth by promoting cholesterol efflux (129). Some drug-resistant ovarian cancer cells rely on fatty acid oxidation (FAO) for energy supply; the CPT1A inhibitor etomoxir enhances chemotherapy sensitivity by blocking FAO (130). Additionally, phospholipid metabolism dysregulation facilitates cancer cell proliferation, and LPCAT inhibitors, which regulate membrane stability, are currently under investigation (131). Although lipid metabolism-targeted therapies remain in clinical research stages, they have shown significant potential in overcoming drug resistance and enhancing immunotherapy efficacy. In the future, these therapies may be combined with PARP inhibitors or immunotherapy to improve treatment outcomes for ovarian cancer patients. Gene Therapy: Gene therapy intervenes in tumor progression by repairing or replacing abnormal genes, demonstrating potential in ovarian cancer research. Current strategies being explored include restoring tumor suppressor genes (e.g., TP53), inhibiting oncogenes (e.g., EGFR), enhancing anti-tumor immunity (e.g., IL-12A/B), targeting angiogenesis (e.g., COL18A1), and increasing drug sensitivity (e.g., BIRC5) (132). In recent years, CRISPR/Cas9 technology has been used for precise editing of BRCA-mutated genes, thereby improving sensitivity to PARP inhibitors, while siRNA/shRNA techniques can suppress drug resistance-associated genes to restore apoptotic signaling (133). Additionally, advancements in viral vectors (e.g., VB-111) and nanoparticle delivery systems have improved the targeting efficiency of gene therapy. Despite its promising potential, gene therapy faces challenges in clinical translation due to low delivery efficiency, biosafety concerns, and the heterogeneity of ovarian cancer. Further optimization and exploration of combinatory applications with existing therapies are needed (134). Cell Cycle Checkpoint Kinase Inhibitors: Cell cycle checkpoint kinase inhibitors ( (5)), including ATR (ceralasertib), CHK1 (prexasertib), and WEE1 (adavosertib), are emerging targeted therapies currently under extensive investigation in clinical trials. The ATR inhibitor ceralasertib enhances the efficacy of PARP inhibitors in homologous recombination deficient (HRD) ovarian cancer by inhibiting the DNA damage response (DDR) pathway (NCT03462342) (124–135). The CHK1 inhibitor prexasertib primarily affects the G2/M checkpoint and may enhance chemotherapy sensitivity in TP53-mutant ovarian cancer (136). The WEE1 inhibitor adavosertib disrupts the G2/M checkpoint, promoting apoptosis in DNA-damaged cancer cells, and has demonstrated efficacy in recurrent TP53-mutant ovarian cancer (137). These inhibitors hold promise for overcoming PARP inhibitor resistance, and future studies may focus on optimizing combination strategies with existing therapies to improve treatment outcomes for ovarian cancer. (**Table 1**: Ongoing Clinical Studies on Other Targeted Therapies for Ovarian Cancer in the Past Five Years).

**Table 1:** Ongoing clinical studies on other targeted therapies for ovarian cancer in the past five years.

Drug Types	Experiment Name	Tumor Type	Treatment regimen	Number of recruits	Primary endpoint	First publication time	References
Tyrosine Kinase Inhibitors (TKIs)	NCT05815862 NCT05145218	Patients with Advanced Lung and Ovarian Cancer Recurrent Platinum- Resistant Ovarian Cancer	AL2846 capsule TQB2450 injection, Anlotinib hydrochloride capsule, and Paclitaxel	40 405	ORR and PFS PFS and OS	2024-12 2024-12	(138) (139)
	NCT04068974	Recurrent Platinum- Resistant Ovarian Cancer	Carrelizumab + Apatinib	28	OFS	2022-06-30	(140)
	NCT04608409	Platinumresistant ovarian cancer	Lapatinib + Paclitaxel	15	PFS and doselimiting toxicity	2025-06-30	(141)
	NCT04502602	Platinumresistant ovarian cancer + other solid tumors	Neratinib +Niraparib	45	PFS	2029-05-31	(142)
Lipid Metabolism Inhibitors	NCT05796973 NCT02223247	Ovarian cancer + other solid tumors Solid tumors	Atorvastatin TVB-2640	240 180	Time to cancer progression and time to death Drug toxicity	2027-12-31 2017-06	(143) (144)
Gene-Targeted Inhibitors	NCT03398655 NCT06305299 NCT03916679	recurrent platinumresistant ovarian cancer Patients with recurrent platinumresistant epithelial ovarian cancer Patients with recurrent and treatmentresistant epithelial ovarian cancer	VB-111 + Paclitaxel iC9-CAR.B7-H3 T cells Anti-MESO CAR- T cells	408 26 20	PFS and OS Drug toxicity, PFS,OS Drug toxicity, PFS	2022-07-19 2026-03 2023-04-20	(145) (146) (147)
	NCT05518253	Ovarian cancer + other solid tumors	CD70 CAR-T cells	36	Drug safety and adverse reactions	2025-05-30	(148)
	NCT05225363	Recurrent epithelial ovarian cancer	TAG72-CAR T cells	33	Drug toxicity, adverse events	2027-04-05	(149)
	NCT04562298	Epithelial ovarian cancer	LCAR-M23	15	Drug toxicity	2022-06-07	(150)
Cell Cycle Checkpoint Kinase Inhibitors	NCT05198804 NCT04065269 NCT05548296	Ovarian cancer Gynecological cancer Platinumresistant ovarian cancer,endometrial adenocarcinoma	WEE1 inhibitor ZN-c3 ceralasertib ACR-368	117 174 390	PFS, Drug safety and tolerance Objective response rate Antitumor activity of the drug	2025-05 2026-04 2027-12-31	(151) (152) (153)

**Table 1**: conducted a search in the ClinicalTrials.gov database using “ovarian cancer” as the keyword, combined with tyrosine kinase inhibitors (TKIs), lipid metabolism inhibitors, gene-targeted inhibitors, and cell cycle checkpoint kinase inhibitors for clinical trials. The selection criteria were restricted to the years 2019–2025, excluding withdrawn studies. This table summarizes relevant clinical trials, providing a valuable reference for the future expansion of targeted therapies for ovarian cancer. Continued monitoring of these study outcomes is essential to advancing more precise treatment strategies.

## 8 Conclusion and outlook

The treatment of ovarian cancer is undergoing a paradigm shift, moving from traditional therapeutic approaches to precision-targeted therapies. With a deeper understanding of the molecular biology underlying ovarian cancer, However, resistance to these treatments is becoming increasingly evident with prolonged use, The major mechanisms include secondary mutations, epigenetic modifications, and immune evasion. 1)BRCA1/2 Mutations: BRCA1/2 mutations are critical pathogenic factors in ovarian cancer, and PARP inhibitors have demonstrated significant efficacy in tumors harboring BRCA mutations. However, with prolonged treatment, tumor cells may acquire secondary mutations that restore homologous recombination repair (HRR) function, thereby evading the effects of PARP inhibitors. For instance, secondary mutations in BRCA1/2 have been detected in patients with clinical drug resistance and have been confirmed to restore DNA repair capability, thereby reducing treatment sensitivity. Consequently, predicting and inhibiting these mutations could help delay the progression of drug resistance. 2)Epigenetic Modifications: Epigenetic modifications also play a crucial role in PARP inhibitor resistance, including DNA methylation, histone modifications, and non-coding RNA regulation. For example, during PARP inhibitor treatment, increased DNA methylation in the BRCA1 promoter region can lead to its downregulation, allowing tumor cells to regain DNA repair ability. Additionally, increased histone deacetylase (HDAC) activity has been associated with resistance, while HDAC inhibitors can restore BRCA1 expression, thereby enhancing tumor sensitivity to PARP inhibitors. Therefore, targeting epigenetic modifications may offer a novel strategy to overcome drug resistance. 3)Immune Evasion Mechanisms: Immune evasion plays a pivotal role in ovarian cancer resistance, particularly affecting the efficacy of immune checkpoint inhibitors (ICIs). Tumor cells can suppress Tcell function and reduce immunotherapy efficacy by upregulating PD-L1 expression and enhancing the immunosuppressive microenvironment, such as the accumulation of myeloid-derived suppressor cells and tumor-associated macrophages. Furthermore, impaired antigen presentation, including reduced tumor antigen expression or the loss of T-cell receptors, can weaken the immune response. To overcome immune evasion, combination therapy has emerged as a key approach. For instance, the combination of ICIs with PARP inhibitors or anti-angiogenic agents can activate the immune system through multiple pathways, thereby enhancing antitumor effects.

Furthermore, with the continuous advancement of personalized medicine, ovarian cancer treatment is increasingly reliant on biomarker-driven therapeutic strategies. BRCA1/2 mutations are among the most important and extensively studied biomarkers in ovarian cancer. Patients harboring BRCA1/2 mutations exhibit a significant therapeutic response to PARP inhibitors. Additionally, the homologous recombination deficiency (HRD) scoring system, which integrates the assessment of BRCA1/2 mutations, loss of heterozygosity (LOH), and tumor mutational burden (TMB), has further expanded the population eligible for PARP inhibitor therapy. As a result, BRCA1/2 mutations and HRD scoring have become indispensable tools in clinical practice for guiding PARP inhibitor use. Other biomarkers have also demonstrated potential in ongoing research. For example, mutations in homologous recombination repair-related genes (HRRm) are closely associated with the efficacy of PARP inhibitors. Finally, circulating tumor DNA (ctDNA) analysis, a liquid biopsy technique, enables real-time monitoring of tumor genetic alterations and resistance mechanisms. ctDNA not only facilitates treatment response evaluation but also aids in the identification of resistance-associated mutations, allowing for timely therapeutic adjustments.

Despite the significant progress in targeted therapy for ovarian cancer, multiple challenges remain to be addressed. Future research should focus on the following areas to further enhance treatment efficacy and address current unmet needs. 1)The Role of Artificial Intelligence (AI) and Bioinformatics in Targeted Therapy: Through big data analysis and machine learning, AI can identify potential therapeutic targets and biomarkers, optimize personalized treatment strategies, and accelerate the development of new drugs. The application of AI contributes to improving treatment precision, reducing toxicity, and expediting clinical trials. 2)The Role of Liquid Biopsy and Circulating Tumor DNA (ctDNA) in Monitoring Treatment Response: Liquid biopsy, particularly circulating tumor DNA (ctDNA) analysis, has emerged as a crucial tool for evaluating therapeutic response. Compared to traditional tissue biopsy, liquid biopsy offers a non-invasive and real-time monitoring approach, facilitating the identification of resistance mutations and timely adjustment of treatment strategies to improve efficacy. The integration of AI-driven dynamic analysis of ctDNA holds promise for further optimizing targeted therapy regimens. 3)Epigenetic Regulators and Antibody-Drug Conjugates (ADCs):Emerging therapeutic targets, including epigenetic regulators and antibody-drug conjugates (ADCs), have demonstrated potential in ovarian cancer treatment. Epigenetic modulators, such as histone deacetylase (HDAC) inhibitors and DNA methylation inhibitors, have shown promise in clinical trials when combined with existing therapies. ADCs, by precisely delivering cytotoxic drugs, reduce adverse effects and enhance therapeutic efficacy, making them a promising approach for future ovarian cancer treatment.

In conclusion, targeted therapies for ovarian cancer are entering a promising new era. With continued research and innovation, there is hope for transforming ovarian cancer into a manageable chronic disease, offering patients longer survival and a better quality of life. As the field progresses, future studies will drive improvements, offering a brighter outlook for ovarian cancer patients.
